# Prediction of surgery start for automated anesthesia using draping detection from surveillance videos

**DOI:** 10.1007/s10877-025-01314-x

**Published:** 2025-06-20

**Authors:** Akihito Ito, Sho Mitarai, Kazumasa Kishimoto, Chang Liu, Goshiro Yamamoto, Yukiko Mori, Moritoki Egi, Tomohiro Kuroda

**Affiliations:** 1 Department of Anesthesia, Kyoto University Hospital, Kyoto, Japan; 2 Department of Human Health Sciences, Graduate School of Medicine, Kyoto University, Kyoto, Japan; 3 Division of Medical Information Technology and Administration Planning, Kyoto University Hospital, Kyoto, Japan; 4 Department of Real World Data R&D, Graduate School of Medicine, Kyoto University, Kyoto, Japan; 5 Preemptive Medicine and Lifestyle Related Disease Research Center, Kyoto University Hospital, Kyoto, Japan

**Keywords:** Anesthesia, Automated anesthesia, Video analysis, Surveillance videos, Event detection

## Abstract

One of the primary goals of automated anesthesia is to reduce human intervention and reduce the workload of anesthesiologists. However, switching modes before the start of surgery still requires manual operation. The present study aims to develop a system that predicts the start of surgery by analyzing the actions of medical staff in the operating room using surveillance camera footage, thereby enabling automated mode transitions in anesthesia systems. We analyzed 110 surveillance videos of elective laparoscopic surgeries at Kyoto University Hospital. Key medical staff actions to predict the start of surgery were identified, and the time intervals between each action and skin incision were recorded. We then developed a detection system to identify draping, the best key action, and evaluated it by comparing system-detected draping times with manually annotated times in 96 videos. Five key actions were identified: hand washing, sterilization, light activation, bed cradle set-up, and draping. The start of draping had the shortest median time interval to the skin incision (7.71 min, interquartile range: 5.89–9.72), which was significantly shorter than that of the other actions (*p* < 0.05), and also had the shortest interquartile range. In the system evaluation, the median time error for detecting draping was 19.0 s (interquartile range: 16.0–50.0). The start of draping is a reliable predictor of the start of surgery, and the draping detection system demonstrated high accuracy. These results support advances in anticipatory automated anesthesia systems, enhancing workflow efficiency and patient safety in the operating room.

## Introduction

One of the characteristics of an anesthesiologist’s work is multitasking because they must simultaneously perform anesthesia procedures, administer medications, maintain records, and sometimes provide education. Frequent switching between these tasks has been suggested to lead to errors [1], potentially compromising patient safety. Automated anesthesia delivery systems have been developed to reduce this burden on anesthesiologists [2, 3]. Closed-loop control systems are commonly used to maintain a stable anesthesia level, and several randomized controlled trials have demonstrated their safety and efficacy during surgeries [4, 5]. Further functional enhancements, such as the administration of vasopressors and fluid therapy, have been developed [6, 7] and broader clinical applications are anticipated. In Japan, companies started to promote automated anesthesia systems from 2023, and the Japanese Society of Anesthesiologists has published guidelines outlining operational standards for both facilities and physicians [8].

However, the current closed-loop control system primarily focuses on improving the intraoperative management of anesthesia, with minimal attention given to transitions between the automated mode and others during anesthesia induction and emergence (such as manual or sequential modes). The necessity for anesthesiologists to manually switch modes has been largely overlooked. The automation of these transitions may reduce the cognitive burden associated with multitasking during anesthesia; however, this remains a significant technical challenge. Closed-loop control systems rely solely on vital signs as input, making mode transitions unachievable through closed-loop control alone. While integrating the anesthesia recording system and using surgical start and end times as triggers for mode switching appears to be feasible, practical limitations complicate this approach. For example, preemptive actions, such as increasing analgesic dosages or discontinuing muscle relaxant administration, must be initiated before surgery begins or ends. Consequently, mode transitions must occur well in advance of the surgical start or end, making this method impractical. To enable the effective automation of mode transitions, a system that is capable of monitoring task progression in the operating room and predicting surgical start and end times in advance is essential.

Previous studies examined the use of laparoscopic video data to recognize intraoperative progress [9–12]. However, laparoscopic videos are only available after surgery has begun, limiting their use to intraoperative applications. Additionally, they cannot be applied to procedures where laparoscopy is not used, limiting their generalizability in predicting the end of surgery. To address these limitations, we propose utilizing surveillance camera footage from operating rooms to track surgical progress. Unlike laparoscopic videos, surveillance footage captures the entire process—from patient entry to exit—regardless of the surgical type. This comprehensive coverage makes it well-suited for monitoring progress across a broader timeframe, including periods before and after active surgery. Another potential approach for recognizing operating room activities is audio-based recognition. However, the content and timing of spoken communication can vary significantly between individuals, and many actions are performed without verbalization. From the standpoint of generalizability, video data offer a more consistent and universal means of capturing operative activities. Therefore, we chose to use video data.

In the present study, we aimed to develop a system that predicts surgical start times in advance. The period leading up to the start of surgery is characterized by a higher density of tasks than in other phases, including the adjustment and fixation of patient positioning aids, the documentation of anesthesia induction and intubation, and the administration of preoperative antibiotics. Therefore, contributing to the automation of mode transitions during this critical phase is significant. From a technical perspective, while the final stage of surgery primarily involves detailed work by the surgeon, the period from patient entry to the start of surgery is characterized by the dynamic activities of all healthcare providers, including anesthesiologists, nurses, and surgeons, as they prepare for the procedure. These dynamic actions are more likely to be captured and recognized by machine-based systems. As a first step, we observed healthcare providers’ actions to establish how much time remained until the procedure began and identified the best action to use as a trigger for predicting the surgical start time. We then developed a system to detect this action and evaluated its accuracy.

## Methods

### Video analysis for identifying medical staff’s actions

To develop a system that predicts the initiation of surgery in advance using operating room surveillance camera footage, it is essential to identify preoperative events that serve as predictive triggers. Studies regarding the use of surveillance videos have suggested that surgical progress can be inferred from operating room video footage [13]. However, they did not identify which specific events in operating rooms may serve as reliable triggers for predicting the start of surgery. While other previous studies measured the interval between patient entry and surgical start [14, 15], their objective was not to predict the surgical start time, and they did not include time measurements of diverse staff behaviors as potential predictors. Therefore, we first identified and listed characteristic actions performed by healthcare providers and compared the time intervals from these actions to the start of surgery. We then identified the action with the smallest median and least variability to select the most reliable predictive trigger.

#### Analysis design

This was a retrospective study performed at a single center, Kyoto University Hospital, Japan. Elective laparoscopic surgeries, including robotic surgeries, performed on patients aged 18 years or older by the Department of Gastrointestinal Surgery or Hepatic Surgery between April 2024 and June 2024 were included, and their surveillance videos were analyzed. We selected this study period that would ensure a sample size of approximately 80 to 100 patients in our hospital, even after excluding following cases, based on reference patient numbers in prior studies on phase recognition and action recognition [9]. Since variability in the time interval from each healthcare provider’s action to the start of surgery was anticipated due to differences in surgical departments and operative sites, this analysis was limited to laparoscopic abdominal surgeries performed in these two surgical departments. Surgeries involving any invasive procedures prior to skin incision (e.g., epidural anesthesia, peripheral nerve block, and central venous catheterization) were excluded because these procedures result in delays. Surveillance cameras were installed on the ceiling in the corners of the operating rooms, capturing a wide-angle view of the entire room. Videos were recorded in HD resolution (1280 × 720 pixels) at a variable frame rate with an average of 30 fps. The surveillance cameras were not equipped with audio-recording capabilities, and only video data were available.

#### Data collection

We reviewed ten surveillance videos and identified medical staff behaviors that may serve as triggers for predicting the start of surgery. We randomly selected ten cases, ensuring that all four subgroups—based on the department (gastrointestinal surgery or hepatobiliary surgery) and the presence or absence of robotic assistance—were represented. We prioritized behaviors that were commonly observed across these ten surgeries and visually identified in surveillance videos. We then recorded the timing of each behavior and the skin incision in all videos. The researcher responsible for this data collection was an experienced anesthesiologist with sufficient expertise to accurately recognize each medical staff member’s actions.

#### Analysis

The time interval between each medical staff’s action and the skin incision was calculated. Median, quartiles, mean, and standard deviation were reported. We used the Wilcoxon signed-rank test, a non-parametric test for paired samples, to compare the distributions of time intervals between the action with the smallest median and each of the other actions. As a subgroup analysis, we performed stratification based on the surgical department (gastrointestinal surgery or hepatobiliary pancreatic and transplant surgery) and whether a robot-assisted procedure was being performed. Median and quartiles were reported, and the same analyses were conducted within each subgroup. In this analysis, a Bonferroni-adjusted p-value of < 0.05 was considered to be significant. Statistical analyses were conducted using scikit-learn (version 1.2.2) in Python (version 3.10.11).

### Evaluation of draping detection system

**Fig. 1:**
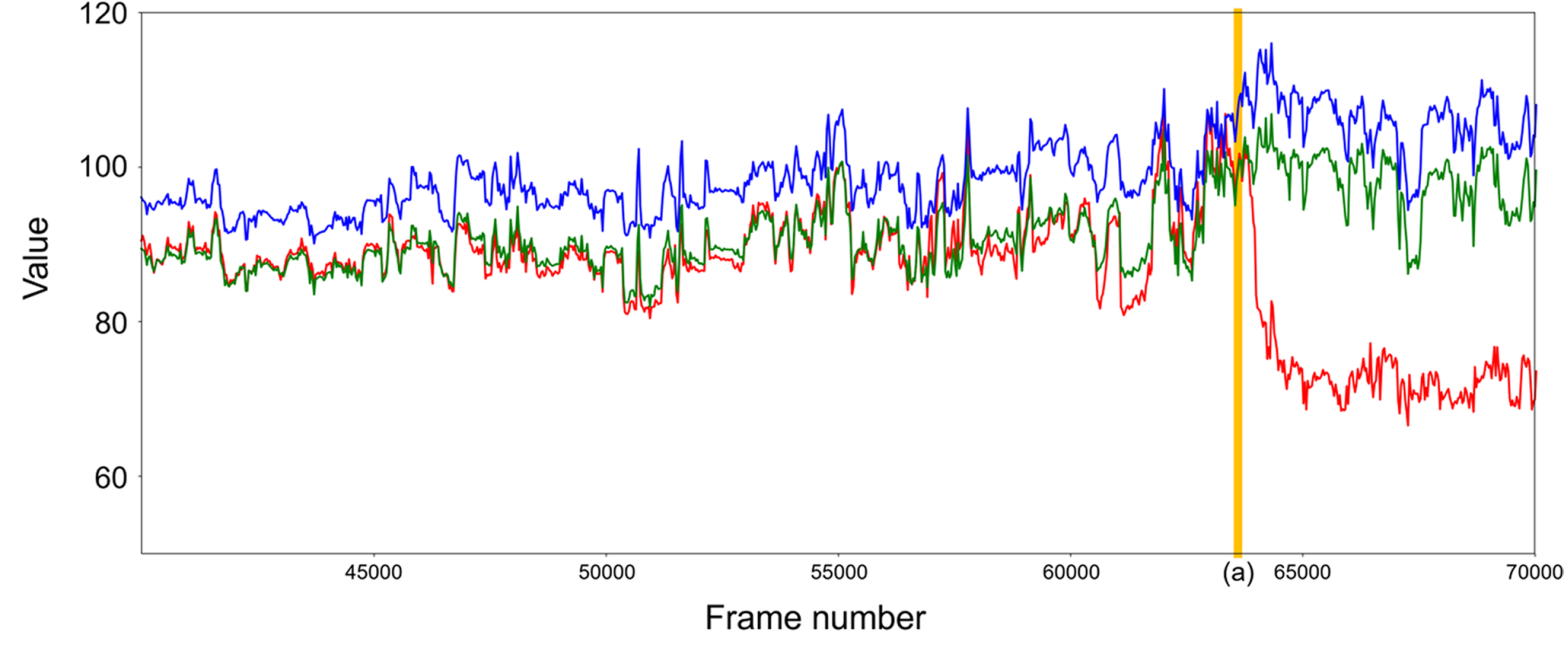
Temporal changes in average color values in the detected bed area. Red, green, and blue values are plotted with each color. Draping started in frame 63,600 (vertical line (**a**)). After draping, the red value was significantly lower than the green and blue values

Based on the results obtained, we developed a system to detect the best action, the draping of a patient by surgeons. Since the sterile drapes used in surgery are distinctively blue, we implemented a detection method that utilizes color information from surveillance camera footage as a key feature. Our system consists of three steps: bed detection, classification of the bed condition, and the identification of draping. In the bed detection step, we use object recognition to detect the surgical bed and crop the video accordingly. Since operating room surveillance cameras capture a wide-angle view of the entire room, information outside the immediate vicinity of the surgical bed—where the main surgical activities occur—is unnecessary. In the classification step, we calculate the average color information of all pixels within the detected bed area and classify the footage as either before or after draping using machine learning. Since the patient is covered with a blue surgical drape, the average pixel color significantly changes after draping (Fig. 1). To capture this change, we employed a Support Vector Machine (SVM), selecting this method for its computational efficiency. In the identification step, we confirm that draping has occurred if the footage is classified as “after draping” for 10 consecutive seconds. This step is implemented to prevent premature false detection caused by misclassification in a few frames. The procedures for each step are described in detail in Sect. 2.2.2. After implementing this system, we evaluated its accuracy.

#### Data used for the evaluation

Inclusion criteria were the same as those in 2.1.1. Additionally, surgeries in which transparent cloths were used for draping and those in which the area around the bed was obstructed by objects in the room (e.g., laparoscopic displays, shadowless lamps, or surgical robotic systems) were excluded.

#### Steps for draping detection

##### Bed detection (Fig. 2)

**Fig. 2:**
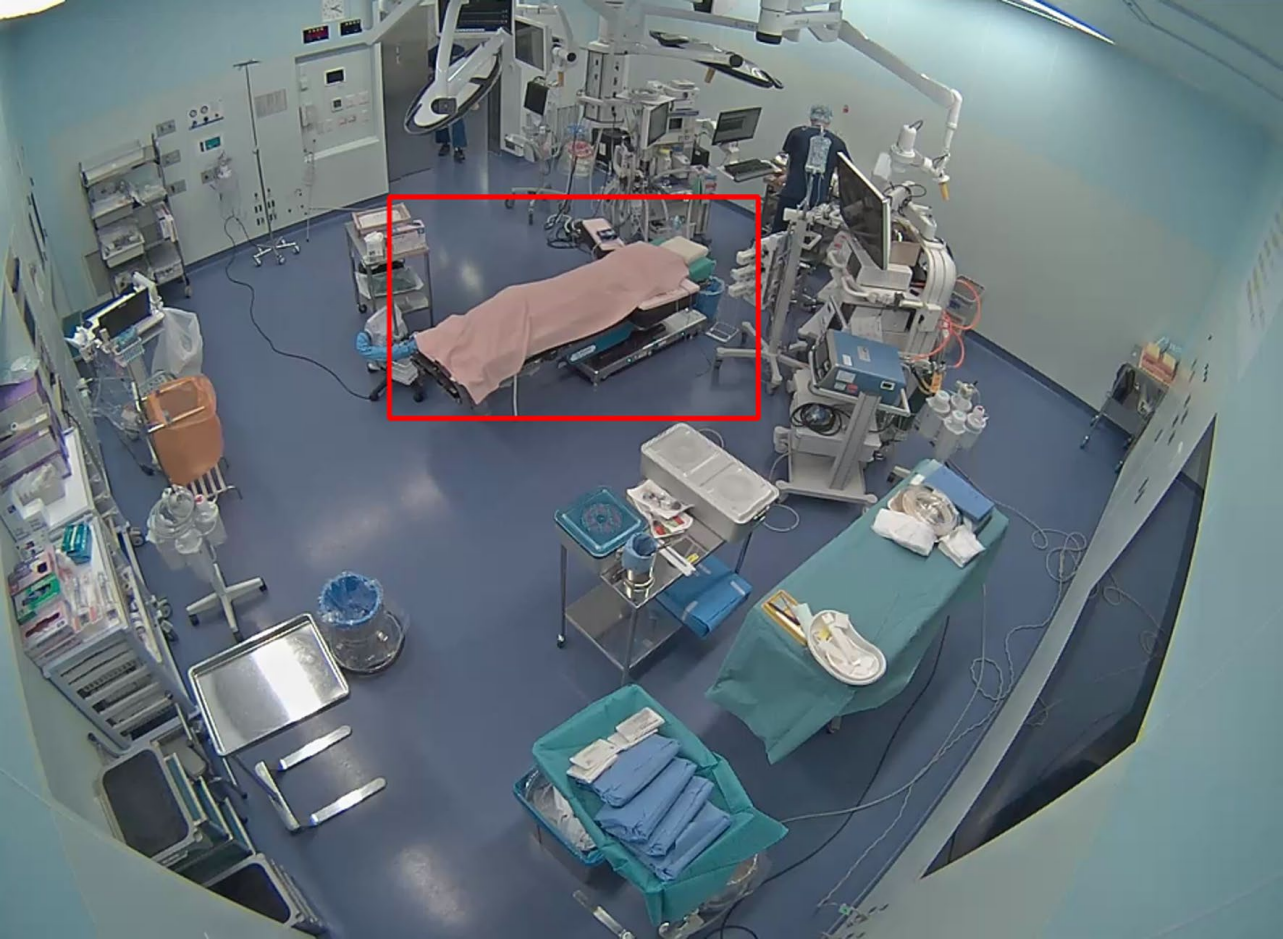
Bed detection in one video. The red bounding box is the operating bed area detected by fine-tuned YOLOv8 and expanded 1.2-fold. This figure showed that bed detection functioned effectively

The surgical bed is detected in the video one minute before the patient enters the operating room. The YOLOv8l model with transfer learning was employed as the detection model [16]. In transfer learning, 350 frames from five videos recorded in different operating rooms at Kyoto University Hospital were utilized. If the bed cannot be detected, the system attempts detection every one minute on five frames prior to the patient’s entrance. The system returns an error when bed detection fails on all attempts. The bounding box area of the detected bed is expanded 1.2-fold to reduce the impact of movements of the bed and is used for the next step.

##### Classification of the bed’s condition

The classification model uses the average color of the bounding box area to establish whether the bed is in the “before draping” or “after draping” condition. After the patient enters, the average color value of all pixels within the bounding box area is calculated every 30 frames (nearly every one second). In addition to the red, green, and blue values of the average color, a custom “cyan-like value” is calculated, where G is the Green value, B is the Blue value, and R is the Red value.

$${\textrm{Cyan-like value}}\; = (G + B) \div 2 - R$$

SVM, using the red, blue, and green values and the cyan-like value normalized to the time of patient entry, classifies each frame as either before or after draping. SVM was configured using an RBF kernel, with other parameters set to their default values of scikit-learn (version 1.2.2). The model was trained on ten surveillance videos in January 2024.

##### Identification of draping

The system identifies draping when ten consecutive frames out of every 30 frames (nearly ten seconds) are all classified as the “after draping” condition.

#### Analysis

In the present study, the researcher visually reviewed all videos and annotated the precise moment of the start of draping. The main outcome measure was the time interval between the system’s identification and the researcher’s annotation. Videos in which the system was unable to detect the draping moment were considered to be failures. The distribution of time intervals was summarized using the median and interquartile range and was visualized with a histogram. In addition, the mean and standard deviation were also reported to provide a complementary summary of the data.

## Results

### Video analysis for identifying medical staff’s actions

**Fig. 3:**
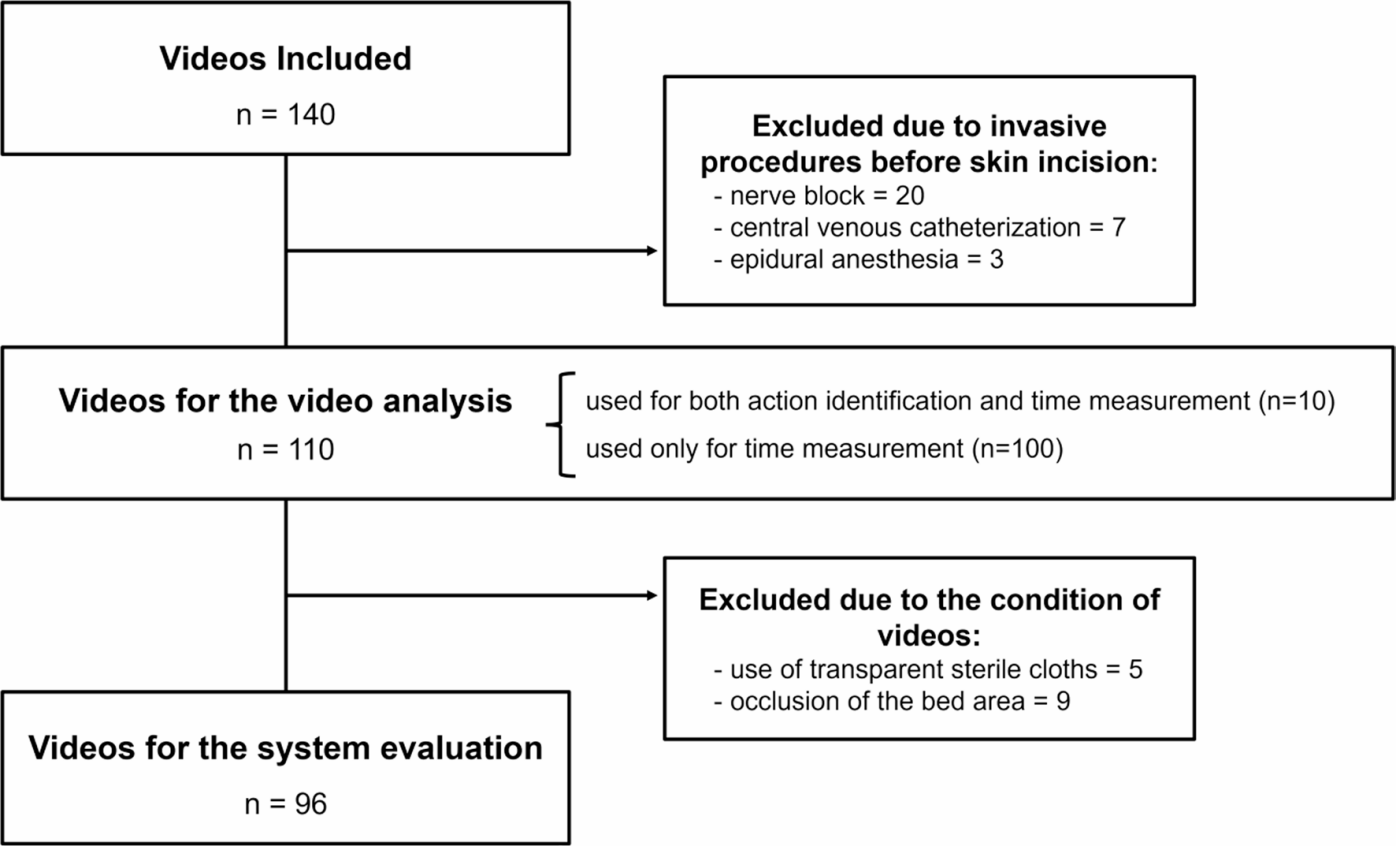
Flow chart of data inclusion and exclusion in this study

A total of 140 videos were eligible for inclusion criteria and thirty videos were excluded (Fig. 3): 20 videos due to nerve block, 7 due to central venous catheterization, and 3 due to epidural anesthesia before the skin incision. Consequently, 110 videos were included in this investigation.

The following five types of medical staff actions preceding surgery were identified as potential triggers for predicting the start of surgery: (1) surgeons returning after washing their hands, (2) the beginning of sterilization, (3) shadowless lamps being turned on, (4) the setting of bed cradles, and (5) the start of draping. The timing of (1) was defined as the moment when the first sterile surgeon entered the room, while the timing of (5) was defined as when the first sterile cloth was attached to the patient’s skin around the abdomen. The surgical time-out by medical staff was also considered a promising trigger candidate. However, it typically occurs only 1–2 min before surgery starts, which is insufficient for an automated anesthesia system to switch modes and adequately raise the patient’s drug effect-site concentration. Therefore, the time-out was excluded in this analysis. Although the completion of intubation was also considered a candidate, it was excluded due to the presence of multiple medical staff around the patient’s head, making it difficult to select the precise timing. Similarly, the preparation of the sterile table by nurses was excluded because it often takes place at the very edge of or sometimes outside the surveillance video’s field of view.

**Fig. 4:**
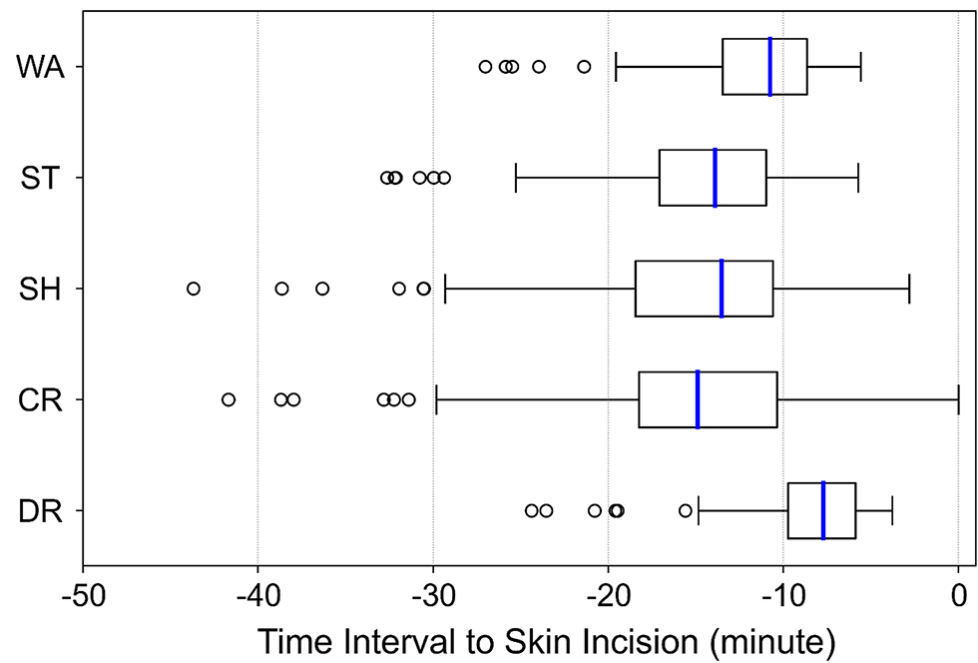
Boxplot of time intervals between each medical staff’s action and the skin incision. The box extends from the first quartile to the third quartile of data, with a blue line at the median. Fliers, data without 1.5× the inter-quartile range (IQR) from the box are plotted as circles. WA: surgeons washing their hands. ST: start of sterilization. SH: turning on the shadowless lamp. CR: setting the bed cradle. DR: start of draping

The time intervals between each medical staff’s action and the skin incision are shown in Table 1 and their distribution is present in Fig. 4. The time interval between the start of draping and the skin incision was 7.71 min (5.89–9.72), which was the shortest median time and interquartile range among the actions investigated. In comparisons with other actions, the start of draping had a significantly shorter time interval to the start of surgery (*p* < 0.05). The results of the subgroup analysis are shown in Tables 2 and 3. In both analyses, which were stratified by department and robot-assisted surgery, the time interval between draping and the skin incision remained the shortest. Notably, in robot-assisted surgeries, the interval between surgeons washing their hands and the skin incision exhibited the least variability, whereas under all other conditions, draping showed the least variability.

**Table 1:** Time intervals between each medical staff’s action and the skin incision. Medians with quartiles and means with standard deviations are shown. (*n* = 110)

Medical staff’s actions	Interval to the skin incision (minute)		Statistical test with draping (Bonferroni adjusted)
	Median (Interquartile range)	Mean (SD)	
Surgeons washing their hands	10.75 (8.64–13.48)	11.83 (4.63)	*P* < 0.0001
Sterilize the patient	13.90 (10.98–17.08)	15.00 (5.65)	*P* < 0.0001
Turn on the shadowless lamp	13.54 (10.59–18.44)	15.14 (7.20)	*P* < 0.0001
Set the bed cradle	14.89 (10.35–18.26)	15.63 (7.36)	*P* < 0.0001
Start of draping	7.71 (5.89–9.72)		-

**Table 2:** Results of the subgroup analysis of surgery departments. The medians and quartiles of the time intervals between each action and the skin incision are shown

Medical staff’s actions	Interval to the skin incision (minute)	
	Hepatic surgery (*n* = 48)	Gastrointestinal surgery (*n* = 62)
Surgeons washing their hands	10.64 (8.04–12.78)	11.14 (8.77–14.04)
Sterilize the patient	16.13 (11.97–19.49)	12.70 (10.72–15.55)
Turn on the shadowless lamp	14.89 (9.73–19.47)	13.04 (10.74–16.28)
Set the bed cradle	16.73 (11.03–20.47)	13.86 (10.35–16.13)
Start of draping	7.08 (5.85–9.62)	8.13 (5.95–10.03)

**Table 3:** Results of the subgroup analysis of the usage of surgery-assist robots. The medians and quartiles of the time intervals between each action and the skin incision are shown

Medical staff’s actions	Interval to the skin incision (minute)	
	Robot-assisted surgery (*n* = 30)	Non-robot-assisted surgery (*n* = 80)
Surgeons washing their hands	13.04 (11.63–15.60)	9.79 (7.88–12.17)
Sterilize the patient	15.19 (12.13–16.85)	13.19 (10.68–18.33)
Turn on the shadowless lamp	15.33 (12.67–18.58)	12.81 (9.73–18.09)
Set the bed cradle	15.88 (12.92–19.75)	14.35 (10.15–17.69)
Start of draping	9.53 (7.95–12.81)	6.90 (5.37–8.99)

### Evaluation of the draping detection system

**Fig. 5:**
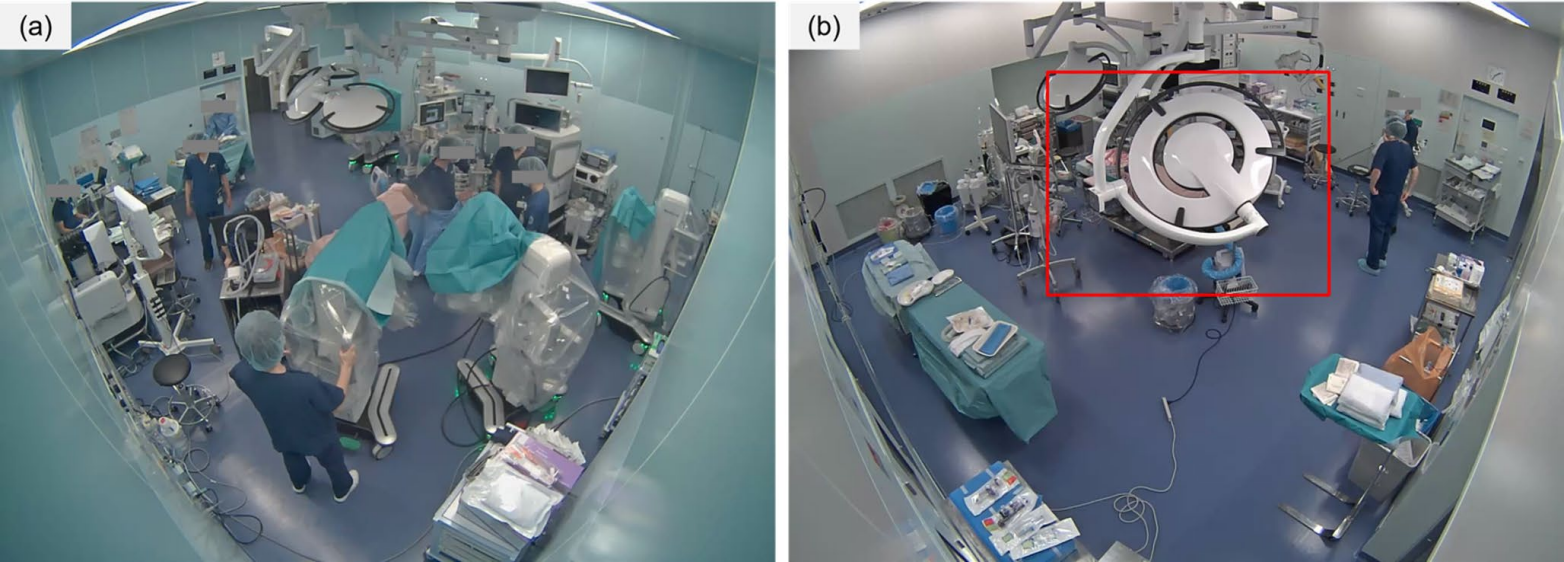
Examples of excluded videos: (**a**) was excluded due to occlusion by surgery-assist robot arms, and (**b**) was excluded due to occlusion by the shadowless lamp. The faces of medical staff are concealed to protect their privacy

**Fig. 6:**
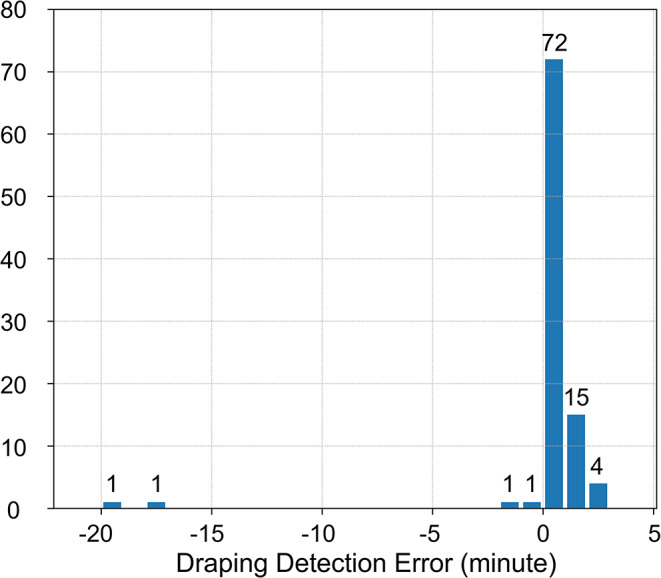
Histogram of time intervals between annotated data and times detected by the system. Negative values mean detection earlier than annotated. Counts are indicated above each bin

To evaluate our system, 96 videos were included in the analysis (Fig. 3). Of the videos available from the video analysis, 5 were excluded due to the use of transparent sterile drapes, while 6 were excluded due to occlusion of the bed area (Fig. 5). The system detected draping in 95 videos (99.0%). The median time error for detected draping was 19.0 s (interquartile range, 16.0–50.0 s) and its distribution is shown in Fig. 6. The mean time error was 11.0 s with a standard deviation of 166.0 s.

## Discussion

The video analysis aimed to identify the most appropriate medical staff action for predicting the start of surgery. In this phase, we listed candidate behaviors that may serve as predictive triggers and measured the time from each action to the initiation of surgery. Among the five extracted behaviors, draping had the shortest time interval to surgery initiation, with the smallest interquartile range. This brevity may be attributed to the inherent sequential nature of surgical procedures, where draping is always performed after handwashing and sterilization. In contrast, in some cases, shadowless lamp activation and bed cradle set-up occurred after draping; however, these actions may be performed at any time between anesthesia induction and surgery initiation, leading to greater variability. Therefore, draping is characterized by its necessity before surgery, its short interval to the start of surgery, and its low variability. The surgical time-out also fulfills these characteristics and may serve as an even more accurate trigger for predicting the start of surgery, provided that it can be reliably detected through audio data or other means. However, from a clinical perspective, the interval from the surgical time-out to the start of surgery is too short, whereas the median interval of 7.71 min interval from draping to the start of surgery is sufficient for adjusting the dosage of analgesic drugs while minimizing unnecessary exposure to deep anesthesia. Based on these findings, draping serves as a reliable predictor for surgery initiation and a suitable trigger for automated anesthesia system adjustments.

In the present study, we limited our analysis to abdominal surgeries. While the five medical staff actions examined are common across various surgical procedures, it remains unclear whether the observed time intervals hold across different specialties. Additional investigations are required before applying draping as a predictive trigger in non-abdominal surgeries. In procedures requiring prolonged preparation, such as cardiac or neurosurgery, the absolute time interval and variability between actions and surgery initiation are expected to be longer. This was also observed in our subgroup analysis, where robotic-assisted surgeries exhibited greater variability. However, the fundamental characteristics of draping—its consistent sequence in the workflow and its short, stable interval to the start of surgery—are likely to remain valid across different surgical fields. Therefore, we anticipate that draping may be adopted as a generalizable predictive trigger for surgery initiation.

Based on the results of the video analysis, we implemented a system for detecting draping and evaluated its accuracy. The system initially performed object detection before patient admission, followed by classification using SVM with color information around the operating bed as features. Xiao et al. reported that medical staff use surveillance camera footage to determine whether surgery is in progress, even when the images are blurred [13]. This implies that color information is important for humans to judge the start of surgery. The mean value of all pixels, which we adopted as a feature, represents highly blurred color information, and thus our method may resemble the process by which humans make their judgments. This relatively simple approach enabled draping detection with a median delay of 19 s relative to manual annotation, and 92.7% of cases were detected within a ± 2-minute margin of error. According to the video analysis, the median time from draping to surgery initiation was approximately 8 min. Given this interval, the detection accuracy of our system is sufficient for preemptively switching modes in an automated anesthesia system or adjusting drug administration before surgery. Twinanda et al. also reported a method for detecting medical staff actions using surveillance camera footage [17]. Their approach utilizes RGBD data to detect various intraoperative procedures and is likely capable of accurately identifying draping events. In contrast, our method focuses specifically on detecting draping, which involves significant color changes. This allows us to forgo depth data and rely solely on widely available RGB surveillance camera footage, providing a practical advantage for draping detection. While incorporating additional features such as audio data or integrating object detection models even after patient admission may improve accuracy, our approach—utilizing a single object detection step and SVM classification at 1-second intervals—achieved adequate accuracy with minimal computational cost. This characteristic lowers the barrier to its implementation in facilities with limited computational resources, making real-time detection more feasible.

The development and validation of this system was conducted exclusively on abdominal surgeries, and its accuracy in detecting draping in other surgical specialties currently remains unclear. One limitation is that our system detects the bed position only before patient admission and does not update it thereafter. Therefore, it cannot be applied to surgeries where the bed orientation changes between patient admission and surgery initiation, such as neurosurgery or otolaryngological procedures. However, since bed repositioning is typically planned in advance, re-detecting the bed position after orientation changes offers a potential solution. Additionally, since our system utilizes color information from the entire area surrounding the bed, it may not be applicable to procedures involving partial draping, such as specific dermatological or plastic surgeries where the entire patient is not covered. In these cases, special adaptations—such as predefining the surgical field area—are required. Despite these limitations, our system is expected to be applicable to a wide range of surgeries that involve full-body draping, even beyond the scope of abdominal surgeries. Further validation is required to confirm its effectiveness in other surgical specialties.

Moreover, since the system was developed and validated using surveillance camera footage from a single institution, its accuracy in other hospitals is unknown. One key factor expected to affect accuracy is the camera angle. If the surveillance camera is positioned in a corner of the ceiling, capturing the entire operating room as in our institution, the system is likely to perform similarly. Even in facilities with different camera angles, the area surrounding the operating bed—the focal point of surgical procedures—is typically within the field of view. Although adjustments specific to each facility may be necessary, the system is expected to be applicable in many institutions. Additionally, since the system relies on color information, its accuracy may not be guaranteed in environments where the drapes or medical staff uniforms closely resemble the patient’s skin tone. Specifically, in facilities using drapes with colors similar to the patient’s skin or uniforms that are visually indistinguishable from the drapes, the system may exhibit frequent misdetections or detection failures, even if trained on facility-specific data. In these cases, the current system alone may have inherent limitations, necessitating the integration of human detection mechanisms or the examination of entirely different approaches that do not rely on color information.

**Fig. 7:**
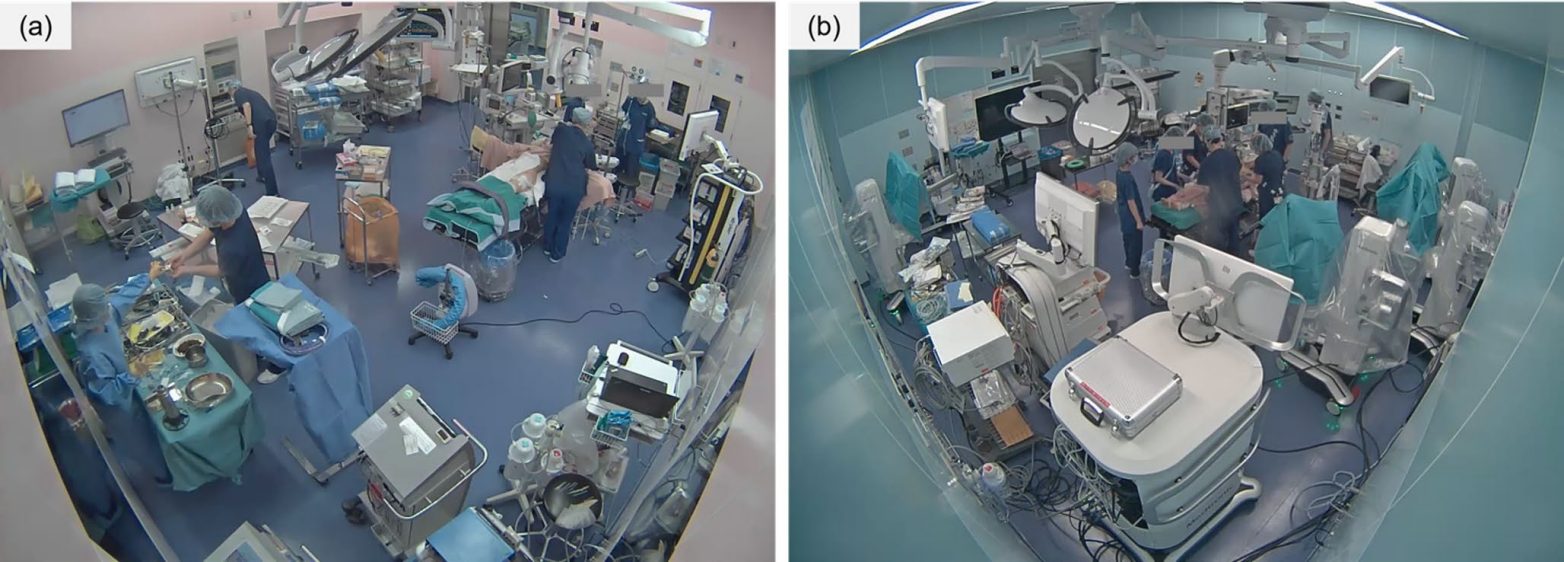
Surveillance videos where draping was detected significantly earlier. In (**a**) and (**b**), the area around the operating bed was occluded by medical staff for several seconds

**Fig. 8:**
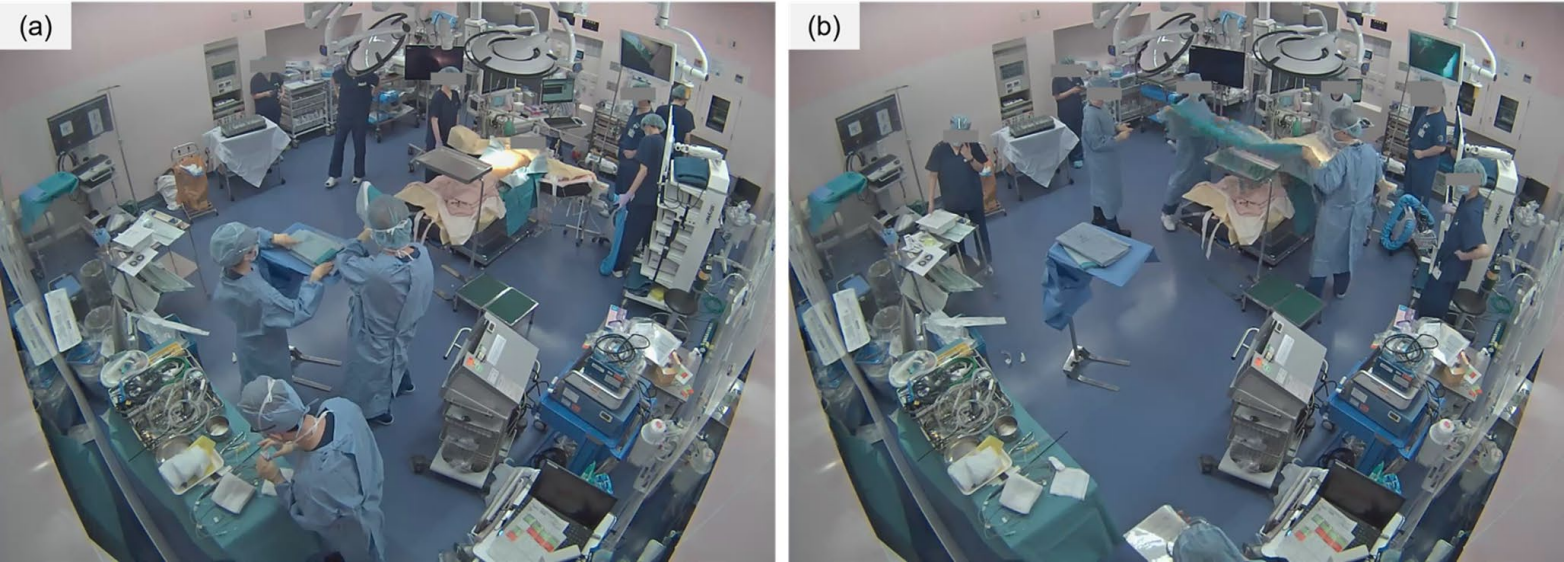
An example of a surveillance video where draping was detected with a delay. After draping began, it took more than one minute to complete the process using a large drape (**a**). The system detected draping when the patient was fully covered with the final drape, one second after (**b**)

Although the developed system successfully detected draping with high accuracy, there were two cases in which it erroneously detected draping at an extremely early stage. These outliers contributed to the large standard deviation observed in the time error. In both cases, prolonged occlusion of the surgical field by medical staff was observed, which likely resulted in misdetection (Fig. 7). Potential countermeasures include integrating human detection to ignore frames with significant occlusion or combining the system with physical triggers, such as the placement of a bed cradle. However, due to the intended application—adjusting anesthetic drug administration or switching anesthesia machine modes before surgery begins—occasional early misdetection may be acceptable. Additionally, there were a few cases in which draping was detected more than two minutes later than the actual start of the procedure. Upon reviewing the footage, we found that this delay was attributed to additional tasks being performed between the initiation of draping and the placement of the large drape covering the patient (Fig. 8). Specifically, in these cases, a small drape was placed at the side of the abdomen before the large drape was used to cover the entire surgical site. In our current definition, draping was marked as initiated when the first sterile cloth adhered to the patient’s skin. Consequently, these cases were classified as delayed detections. However, the system successfully detected the moment when the large drape was applied without delay, suggesting that the timing detected was still sufficient for anesthesia adjustment purposes.

The draping detection system proposed in the present study enables the anticipation of imminent surgical initiation. While identifying the start of surgery is relatively straightforward, such as by linking it with the “surgery start” button in the anesthesia record, the ability to predict it in advance is a novel approach. This capability may be applied to automatically switch an automated anesthesia system to its active mode before surgery begins. Even in cases where an automated anesthesia system is not used, the system may serve as a means to reduce the multitasking burden of anesthesiologists by notifying them in advance that surgery is about to commence, thereby contributing to patient safety. However, the present study evaluated the system’s accuracy using stored video data. Further investigations are required to confirm whether the system functions similarly in real-time processing. Future research needs to focus on confirming its real-time performance and assessing its practical utility in clinical settings.

## Conclusion

We listed medical staff’s actions before the start of surgery and compared the time from each action to the start of surgery using surveillance camera footage of laparoscopic gastrointestinal surgeries. Among the listed actions, the time from the start of draping to the surgical start was the shortest and had the least variability, demonstrating its usefulness as a trigger for predicting the surgical start time in advance. Additionally, we showed that draping was detectable from surveillance camera footage using an object detection model combined with machine learning based on color information around the surgical bed. These results indicate the potential to automate mode transitions in automated anesthesia systems before surgery begins or notify anesthesiologists that surgery is imminent. These applications will help reduce the workload of anesthesiologists and contribute to patient safety.

## Data Availability

No datasets were generated or analysed during the current study.
