# Correction: Prediction of surgery start for automated anesthesia using draping detection from surveillance videos

**DOI:** 10.1007/s10877-026-01428-w

**Published:** 2026-03-12

**Authors:** Akihito Ito, Sho Mitarai, Kazumasa Kishimoto, Chang Liu, Goshiro Yamamoto, Yukiko Mori, Moritoki Egi, Tomohiro Kuroda

**Affiliations:** 1 Department of Anesthesia, Kyoto University Hospital, Kyoto, Japan; 2 Department of Human Health Sciences, Graduate School of Medicine, Kyoto University, Kyoto, Japan; 3 Division of Medical Information Technology and Administration Planning, Kyoto University Hospital, Kyoto, Japan; 4 Department of Real World Data R&D, Graduate School of Medicine, Kyoto University, Kyoto, Japan; 5 Preemptive Medicine and Lifestyle Related Disease Research Center, Kyoto University Hospital, Kyoto, Japan

**Correction to: Journal of Clinical Monitoring and Computing (2026) 40:279–289**

10.1007/s10877-025-01314-x

In this article, the wrong figure appeared in Fig. 6. The incorrect and corrected versions of Figure 6 are provided below:

Incorrect version of Fig. 6:

**Fig. 6:**
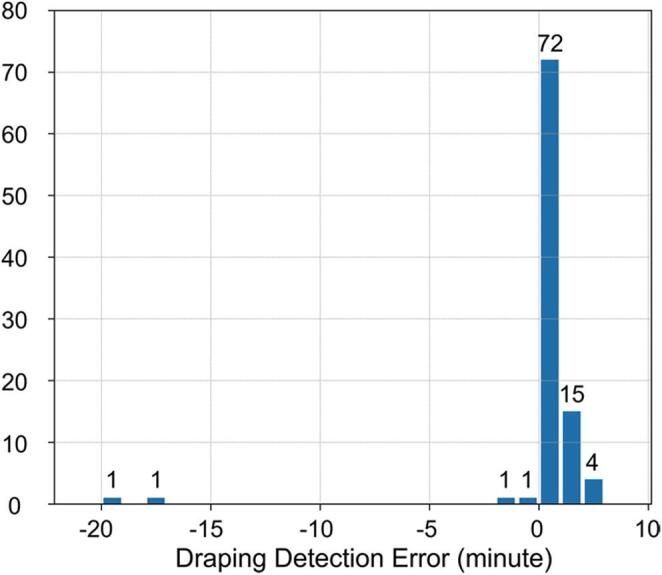
Histogram of time intervals between annotated data and times detected by the system. Negative values mean detection earlier than annotated. Counts are indicated above each bin

Correct version of Fig. 6:

**Fig. 6:**
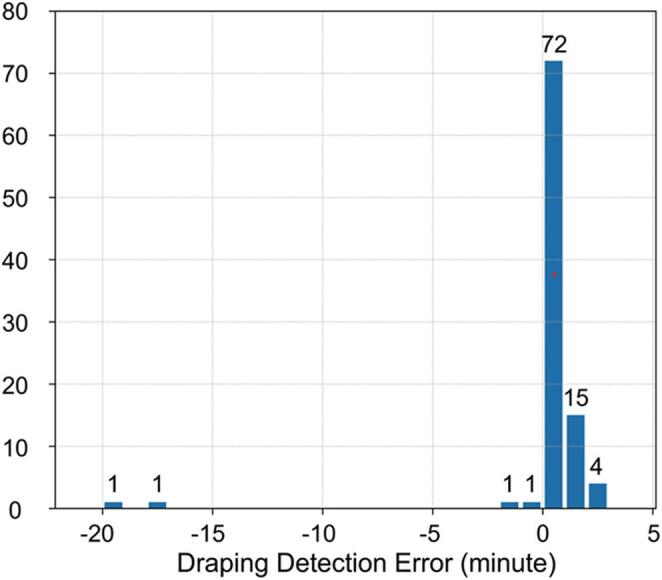
Histogram of time intervals between annotated data and times detected by the system. Negative values mean detection earlier than annotated. Counts are indicated above each bin

The original article has been corrected.

